# Genetic resistance in barley against *Japanese soil-borne wheat mosaic virus* functions in the roots

**DOI:** 10.3389/fpls.2023.1149752

**Published:** 2023-03-10

**Authors:** Kaori Okada, Wenjing Xu, Kohei Mishina, Youko Oono, Tsuneo Kato, Kiyoshi Namai, Takao Komatsuda

**Affiliations:** ^1^ Tochigi Prefectural Agricultural Experiment Station, Utsunomiya, Tochigi, Japan; ^2^ Crop Research Institute, Shandong Academy of Agricultural Sciences (SAAS), Ji’nan, Shandong, China; ^3^ Institute of Crop Science, National Agriculture and Food Research Organization (NARO), Kan-non-dai, Ibaraki, Japan; ^4^ Graduate School of Horticulture, Chiba University, Matsudo, Chiba, Japan

**Keywords:** barley, soil-borne disease, *Polymyxa graminis*, viral pathogenetic, genetic resistance, root

## Abstract

Infection by the *Japanese soil-borne wheat mosaic virus* (JSBWMV) can lead to substantial losses in the grain yield of barley and wheat crops. While genetically based resistance to this virus has been documented, its mechanistic basis remains obscure. In this study, the deployment of a quantitative PCR assay showed that the resistance acts directly against the virus rather than by inhibiting the colonization of the roots by the virus’ fungal vector *Polymyxa graminis*. In the susceptible barley cultivar (cv.) Tochinoibuki, the JSBWMV titre was maintained at a high level in the roots during the period December–April, and the virus was translocated from the root to the leaf from January onwards. In contrast, in the roots of both cv. Sukai Golden and cv. Haruna Nijo, the titre was retained at a low level, and translocation of the virus to the shoot was strongly suppressed throughout the host’s entire life cycle. The roots of wild barley (*Hordeum vulgare* ssp. *spontaneum*) accession H602 responded in the early stages of infection similarly to those of the resistant cultivated forms, but the host was unable to suppress the translocation of the virus to the shoot from March onwards. The virus titre in the root was presumed to have been restricted by the action of the gene product of *Jmv1* (on chromosome 2H), while the stochastic nature of the infection was suppressed by the action of that of *Jmv2* (on chromosome 3H), a gene harbored by cv. Sukai Golden but not by either cv. Haruna Nijo or accession H602.

## Introduction

The *Japanese soil-borne wheat mosaic virus* (JSBWMV), which belongs to the genus *Furovirus* and family *Virgaviridae*, can infect barley (*Hordeum vulgare*), wheat (*Triticum* spp.), and certain other related species. The virus was first detected in 1973 in Japan (Shirako and Brakke, 1984; Shirako et al., 2000). Its genome comprises bipartite positive-strand RNAs, one section being of length 6.5 kb and the other of length 3.5 kb. The virus is transmitted to its host’s root by the plasmodiophorid fungal vector *Polymyxa graminis* (Ye et al., 1999; Ohki et al., 2017). Upon germination, the fungal resting spores develop into zoospores, which, after invading a host root, release virus into the root tissue. In a susceptible host, the virus multiplies in the root, after which it is translocated to the leaf *via* the host’s vascular system. Colonized leaves display chlorosis and mosaicism, which in severe infections result in stunting and subsequent losses in productivity (Campbell, 1996; Cadle-Davidson et al., 2006; Kuhne, 2009). Virus particles retained within the vector’s resting spores can remain infective for many years (Braselton, 1995; Xu et al., 2018). A number of agronomic interventions have been attempted, including crop rotation, late sowing, and chemical control of *P. graminis*, but none has proven to be either practical or effective (Braselton, 1995; Kanyuka et al., 2003). Thus, the only effective strategy to combat JSBWMV appears to be to breed cultivars which harbor gene(s) conferring resistance.

The *Furovirus*-type species *soil-borne wheat mosaic virus* (SBWMV) is a pathogen against which several genes for resistance in bread wheat (*T. aestivum*), durum wheat (*T. durum*), and barley are known. *Sbm1*, which confers resistance in bread wheat against both SBWMV and *soil-borne cereal mosaic virus* (SBCMV), has been mapped to a location on the long arm of chromosome 5D (Brakke and Langenberg, 1988; Narasimhamoorthy et al., 2006; Hao et al., 2012); *Sbm2*, which is also effective against both SBWMV and SBCMV, is located on the short arm of chromosome 2B (Bayles et al., 2007). The durum wheat locus *QSbm.ubo-2BS* makes a modest contribution to the level of host resistance against SBCMV, and its genomic site is similar to that of *Sbm2* (Maccaferri et al., 2011; Russo et al., 2012). In barley, JSBWMV resistance is conferred by a major gene present in cultivar (cv.) Haruna Nijo, mapping to a site on chromosome 2H, with a series of minor effect loci present on chromosomes 3H, 5H, and 6H (Okada et al., 2020). The two major genes *Jmv1* (chromosome 2H) and *Jmv2* (chromosome 3H) present in cv. Sukai Golden act together to confer complete resistance (Okada et al., 2022). As yet, the mechanistic basis of none of these resistances has been characterized.

Host resistance against JSBWMV could potentially operate in one (or more) of four ways: firstly, by blocking the exit of the virus from the vector (Singer et al., 2003); secondly, by silencing the viral RNA (Soosaar et al., 2005); thirdly, by inhibiting the virus replication (Hou et al., 2012); and fourthly, by preventing the translocation of the virus from the root to the shoot (Ohki et al., 2019). With respect to SBCMV, a different mode of resistance is exemplified by the bread wheat variety cv. Cadenza, in which viral RNA, but not the viral coat protein (CP), is detected in the root, suggesting the host’s ability to disassemble the viral particles and either inhibit the further synthesis of viral CP or ensure its proteolytic degradation (Lyons et al., 2009). The aim of the present investigation was to quantify the JSBWMV RNA present in the host barley roots and leaves in order to gain an understanding of the viral pathogenesis and show how the products of *Jmv1* and *Jmv2* operate to confer resistance.

## Materials and methods

### Plant materials and growth condition

Barley cv. Haruna Nijo and cv. Sukai Golden are both resistant to JSBWMV, whereas both cv. Tochinoibuki and the wild barley (*H. vulgare* ssp. *spontaneum*) accession H602 are susceptible (Okada et al., 2020; Okada et al., 2022). Both cv. Tochinoibuki and cv. Sukai Golden are two-rowed malting varieties bred at the Tochigi Prefectural Agricultural Experimental Station (Taniguchi et al., 2001; Takayama et al., 2011). The plants were exposed to JSBWMV by growing them in a disease nursery at Yawara (Tsukuba-Mirai, Ibaraki, Japan). The materials were sown on October 21, 2015. The leaf and root tissue of five plants per entry was sampled six times: December 7, January 26, February 24, March 15, and April 19. To act as a negative control, the same set of entries was also grown in sterile soil for 2 weeks. The JSBWMV content of each leaf and root sample of each individual plant was quantified using a TaqMan assay.

### Microscopic detection of *P. graminis*

The presence of *P. graminis* propagules was revealed in roots sampled on February 24, 2016 from plants grown in JSBWMV-contaminated soil. A total of about 50 cm of root (including both primary and secondary roots) was sampled from each plant. The cotton blue staining procedure adopted for visualizing the propagules has been published elsewhere (Liu et al., 2016).

### TaqMan-based real-time PCR assay targeting *P. graminis*

DNA was extracted from 100 mg of fresh roots using DNeasy Plant Mini Kit (Qiagen, Tokyo, Japan) using the suppliers’ protocols. The TaqMan assay used to quantify *P. graminis* was targeted to the rDNA ITS using primers and probes described elsewhere (Liu et al., 2016) and listed in **Table 1**. Each 10-µl reaction contained 2× Sso Advanced Universal Supermix (Bio-Rad, Tokyo, Japan), 10 ng DNA template, 300 nM of either *P. graminis* rDNA ITS or wheat actin primers, and 300 nM of either *P. graminis* rDNA ITS or wheat actin probe. The reaction was initiated by a denaturing (95°C/30 s) step, which was followed by 50 cycles of 95°C/10 s and 55°C/30 s using a CFX96 real-time system device (Bio-Rad). The baseline threshold was set to 100 for both FAM and Texas Red. Two technical replicates were used for each plant, along with three biological replicates per treatment. The Tukey–Kramer method was applied to find multiple-sample means that are significantly different from each other.

**Table 1:** Sequences of the primers and probes used.

Organism/gene	Sequence/PCR/qPCR	Primer or probe	Sequence (5’ –3’)
*Polymyxa graminis*/rDNA ITS	PCR/qPCR	PgxFw5_Y12824_148	TAGTAGACGCAGGTCATCAACC
	PCR/qPCR	PgxRv9_Y12824_322	CTTCTTCTTCCTCTAGTCGTCCA
	qPCR	PgRealP probe	[FAM]-CCGGTGAACAATCG-[BHQ1]
Wheat/actin gene	PCR/qPCR	Actin Fw	GAGAGGAAGTACAGTGTC
	PCR/qPCR	Actin Rv	AGCCAGAATAGATTCAGAA
	qPCR	Actin probe	[TEX615]-AGACAACTCGCAACTTAGA-[IAbRQSp]
JSBWMV/coat protein gene	PCR/qPCR	SBWMV_CP_Fw	GGTGGTGAAGCAGTTATG
	PCR/qPCR	SBWMV_CP_Rv	CAACGTCTGATCTGTCTG
	qPCR	SBWMV_CP_Prove	(FAM)ACTCACGGTAGCACTCCAATCC(BHQ1)
Barley/actin gene	PCR/qPCR	Hvactin_Fw	GTACCTTCCAACAGATGTG
	PCR/qPCR	Hvactin_Rev	CAGACAACTCGCAACTTA
	qPCR	Hvactin_probe	(VIC)TCGCTGGACCTGACTCATCGTA(MGB)

### Cloning and sequencing of the gene encoding the JSBWMV coat protein

Total RNA was extracted from each sample using a Qiagen RNeasy Plant Mini Kit using the suppliers’ protocols then converted to cDNA using SuperScript III reverse transcriptase (Invitrogen, Tokyo, Japan) and SBWMV-UNIR (Clover et al., 2001). TaqMan primers and probe were targeted to the JSBWMV coat protein sequence. PCRs were formulated with PrimeSTAR HS DNA Polymerase (Takara Bio inc., Kusatsu, Shiga, Japan) and the primer pair SBWMV-UNIF/SBWMV-UNIR (Clover et al., 2001). The resulting amplicon (expected size: 338 bp) was verified by separation through a 1.5% agarose gel and purified using a QIA quick PCR Purification Kit (Qiagen) following the manufacturer’s protocol. The purified amplicons were ligated into pUC118 (Takara Bio inc.) and transformed into *E. coli* DH5α competent cells (Invitrogen) for sequencing using a Hitachi 3130X Genetic Analyzer.

### TaqMan assay used to quantify JSBWMV

The RNAs required to conduct a TaqMan assay of JSBWMV were extracted from 100 mg fresh plant material using a Qiagen RNeasy Plant Mini Kit fitted with a column DNase Digestion Kit (Takara Bio inc.) using the suppliers’ protocols. The RNA concentrations were estimated spectrophotometrically and adjusted to 50 ng/µl. The primers and probe used in the TaqMan assay were named, respectively, SBWMV_CP_Fw/_Rv and SBWMV_CP_Prove, and their sequences are given in **Table 1**. The target for both the primers and the probe was the RNA2 region encoding the virus’ coat protein (LC642565.1) (Okada et al., 2022). The sequence of the barley gene encoding actin (HORVU.MOREX.r3.5HG0457850: Genbank accession AK362208.1) was used as the internal control. The forward primer was designed to span an exon–exon junction in order to avoid its annealing to genomic DNA.

Each 6.5-µl reverse transcription reaction contained 150 ng total RNA, 20 nmol dNTP, 5 pmol SBWMV_CP_Rv, 125ng oligo (dT)12-18 primer (Invitrogen, Tokyo), and 0.25 µl RNase free water. The reaction was held at 65°C for 5 min and then quenched on ice before adding 5× First-Strand buffer, 0.5 µl 0.1 M DTT (Invitrogen), 20 U RNaseOUT (Invitrogen), and 100 U SuperScript III reverse transcriptase (Invitrogen). The reactions were incubated for 5 min at 25°C, then for 1 h at 55°C, and then stopped by holding at 75°C for 15 min. The resulting cDNA preparations were diluted by the addition of 15 µl 0.1× Tris-EDTA buffer.

Each 10-µl TaqMan reaction contained 2× SsoFast Probe Supermix (Bio-Rad), 300 nM of the JSBWMV or barley actin primer pairs, 300 nM of either the JSBWMV or the barley actin TaqMan probe, and 6 ng cDNA. The reactions were subjected to an initial denaturing step of 95°C/30 s, followed by 50 cycles of 95°C/10 s, 55°C/30 s, using a Step One System Device (Applied Biosystems, Waltham, MA, USA). Ct values were automatically analyzed by v2.3 StepOne™ software. The baseline threshold was set to 0.1 and 0.15 for each of FAM and Cy5. Two technical replicates were included for each plant, along with five biological replicates for each treatment. Welch’s *t*-test for unequal variances was applied to see if two sample means are significantly different.

## Results

### *P. graminis* colonizes the roots of both JSBWMV-resistant and JSBWMV-susceptible barley varieties

**Figure 1:**
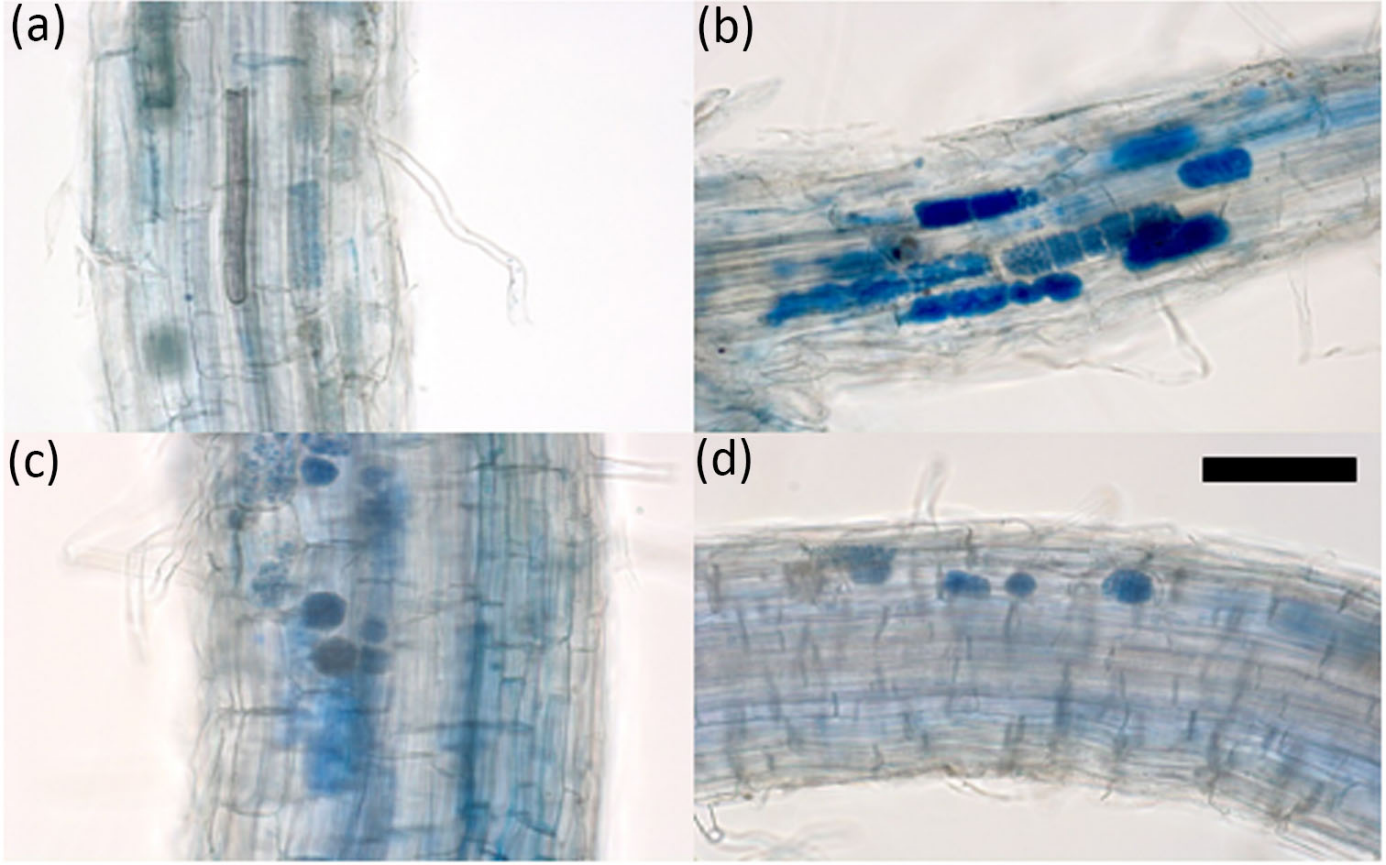
Detection of *P. graminis* colonization of barley root. Roots of **(A)** cv. Tochinoibuki, **(B)** cv. Sukai Golden, **(C)** cv. Haruna Nijo, and **(D)** wild barley accession H602. Plants were grown in a JSBWMV-infested field at Yawara (Tsukubamirai, Japan). Samples were collected in late February 2016. The cotton blue staining procedure followed that described by Liu et al. (2016). Scale bar: 100 μm.

**Figure 2:**
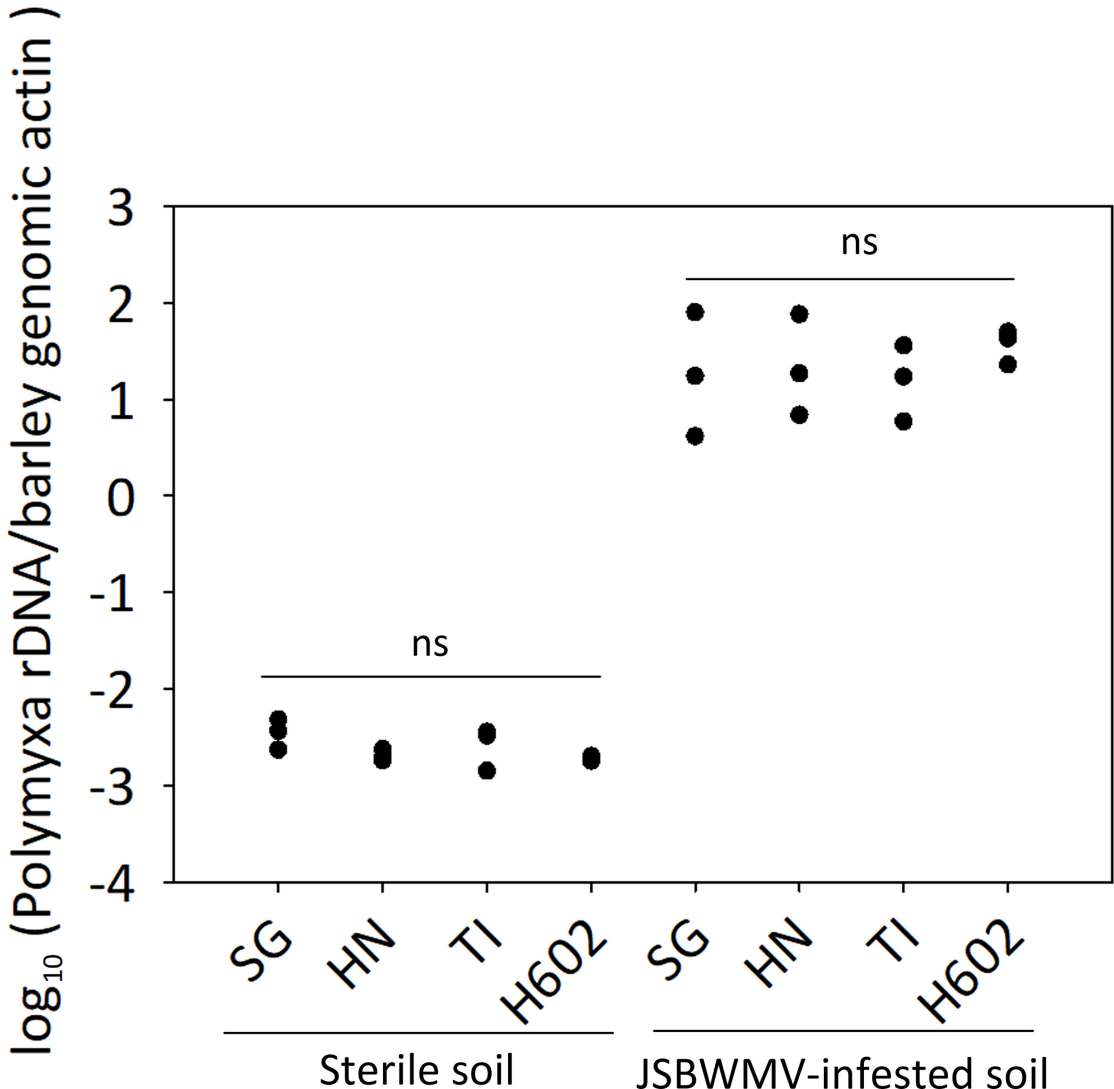
*P. graminis* propagule copy number in the roots of barley plants grown in sterile soil or in JSBWMV-infested soil. The copy numbers were deduced using a quantitative PCR assay. TI, cv. Tochinoibuki; SG, cv. Sukai Golden; HN, cv. Haruna Nijo; ns, non-significant (*p* > 0.05) by Tukey–Kramer method (*n* = 3). The standard curves used to deduce absolute quantities are shown in **Supplementary Figure S1**.

Cotton blue staining of the roots developed by cv. Tochinoibuki, cv. Sukai Golden, and cv. Haruna Nijo, as well as of accession H602, when grown in JSBWMV-infested soil, showed that *P. graminis* was able to successfully colonize the roots in each case: Mature sporosori containing large numbers of resting spores were visible within the root cortex cells (**Figure 1**). Thus, the JSBWMV resistance exhibited by both cv. Sukai Golden and cv. Haruna Nijo (Okada et al., 2020; Okada et al., 2022) cannot be due to the host’s ability to suppress *P. graminis* colonization. The quantitative PCR assay of infected roots suggested the absence of any significant (Tukey–Kramer method) variation in the number of vector spores present in the host roots, which further supported the notion that the hosts’ JSBWMV resistance, rather than inhibiting the entry of the vector, results from the suppression of the virus’ *in planta* multiplication (**Figure 2**).

### Estimating the JSBWMV titre *in planta*

**Figure 3:**
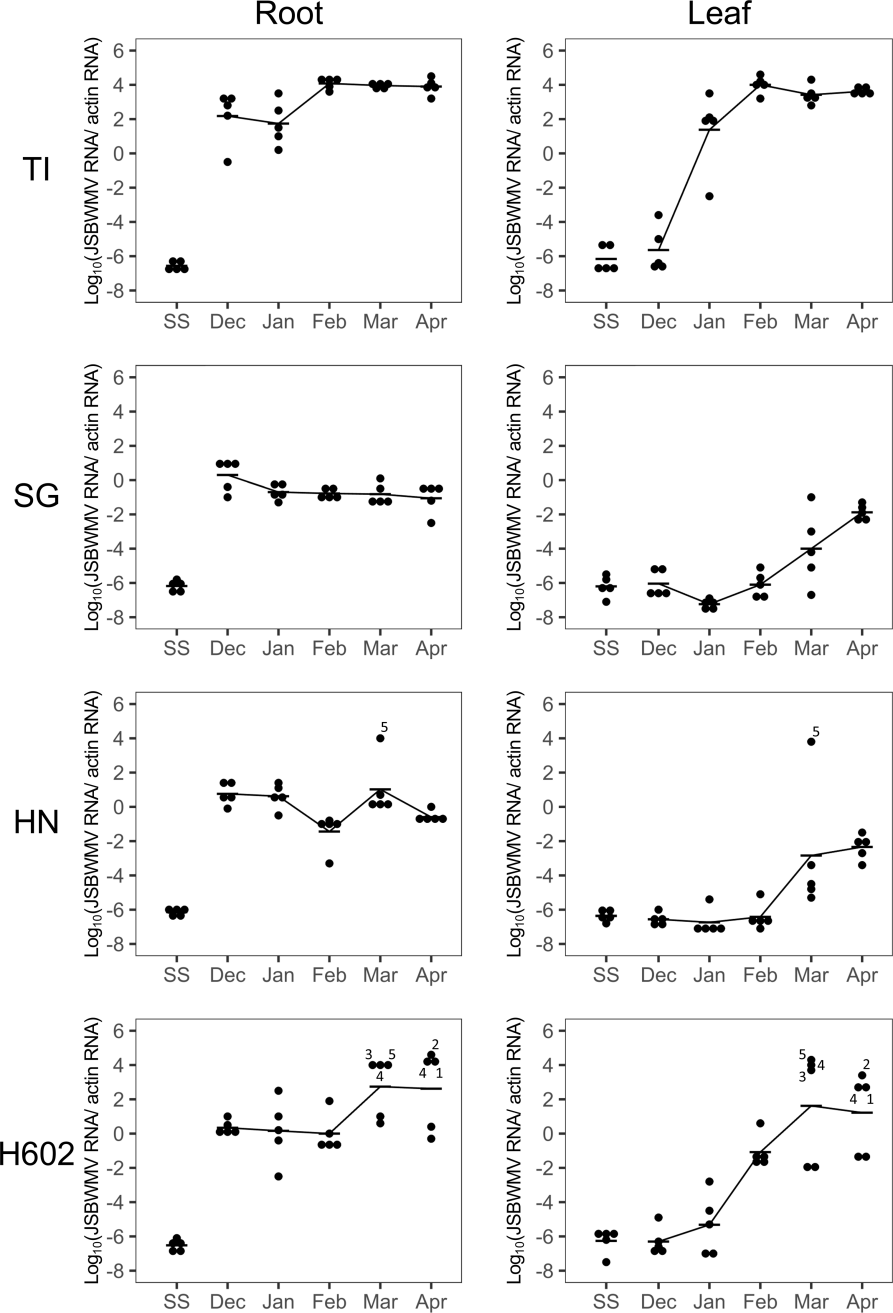
Quantification of JSBWMV present in the root and leaf of plants grown in a virus-infested field. TI, cv. Tochinoibuki; SG, cv. Sukai Golden; HN, cv. Haruna Nijo; SS, sterile soil. In HN and H602, plants with virus titre score >2 were numbered. Data shown in the same number refer to the root and leaf material sampled from the same plant at each given sampling time.

The JSBWMV sequence of the gene-encoding coat protein was the same as reported previously (Okada et al., 2022). The TaqMan probe and primers produced against the sequence were used for the TaqMan quantitative assay of JSBWMV (**Supplementary Table S1**). From December onwards, the viral titre in the roots of susceptible cv. Tochinoibuki plants grown in virus-infested soil was significantly higher (*t*-test, *p* < 0.01) than that in the roots grown in sterile soil (**Supplementary Table S2**), showing that the virus had already begun multiplying *in planta* at this time (**Figure 3**). By January, the viral titre in the leaves of diseased cv. Tochinoibuki plants was higher than in the non-exposed plants’ leaves, demonstrating that the process of root–leaf translocation was already underway. The viral titre in both the root and leaf peaked in February and remained stable until April, by which time the plants of each of the three cultivars had almost reached physiological maturity, while those of accession H602 were close to anthesis.

In the roots of cv. Sukai Golden plants, the viral titre was consistently lower (*t*-test, *p* < 0.01) (**Supplementary Table S2**) than that measured in infected cv. Tochinoibuki roots throughout the period January to April. The titre in cv. Sukai Golden leaf tissue was significantly higher (*t*-test, *p* < 0.01) than in the non-exposed plants’ leaf tissue but significantly lower (*t*-test, *p* < 0.01) than in cv. Tochinoibuki leaf tissue all the way from January to April. The implication is that the resistance displayed by cv. Sukai Golden acted by suppressing the replication of the virus in the root. The behavior of cv. Haruna Nijo plants was largely similar to that of cv. Sukai Golden plants. During March and April, a lower proportion of cv. Haruna Nijo plants showed a titre above 3.0 in their root or leaf tissue than was the case for accession H602 plants.

**Figure 4:**
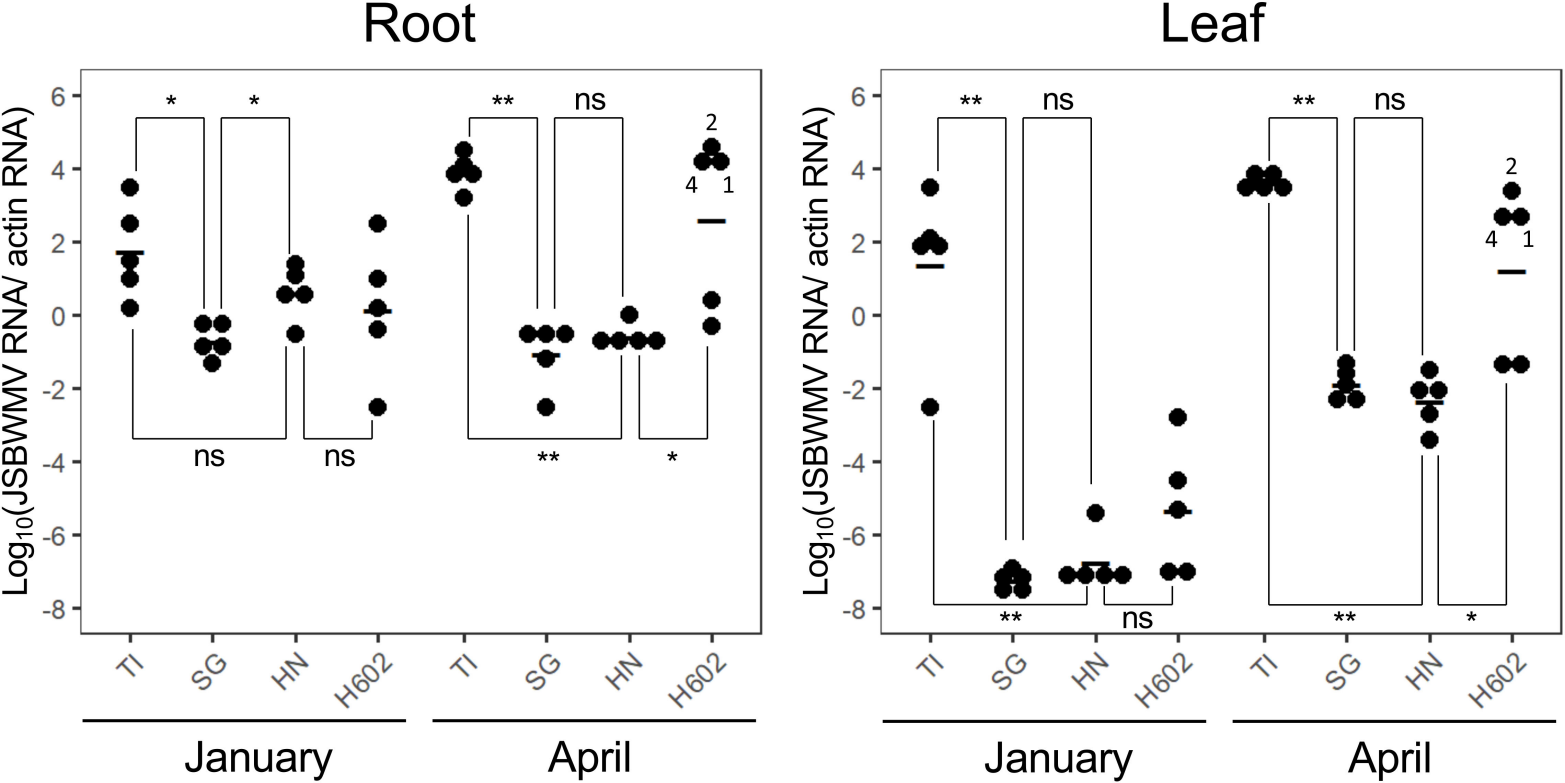
Shifting quantity of JSBWMV present in the root and leaf of plants grown in a virus-infested field. TI, cv. Tochinoibuki; SG, cv. Sukai Golden; HN, cv. Haruna Nijo. *, significant at 5% probability level by Welch’s *t*-test; **, significant at 1% probability level by Welch’s *t*-test (*n* = 5).

The viral titre in the roots of accession H602 plants was not as high as in cv. Tochinoibuki roots during December and January; rather, it matched the level seen in the roots of cv. Haruna Nijo and cv. Sukai Golden plants (**Figures 3**, **4**). The inference was that the mode of resistance during the early part of the host’s life cycle was similar in accession H602 and the two resistant cultivated barleys. However, as the season progressed through March and April, the titre in both the root and leaf of some individual plants of accession H602 diverged from that seen in cv. Sukai Golden and cv. Haruna Nijo plants, although it remained at the level of the two resistant cultivars in some other individuals.

## Discussion

### Genetic resistance in barley to JSBWMV

JSBWMV relies on its vector *P. graminis* to gain entry into its host plant. Extensive investigations, based on either microscopy and/or PCR, have so far failed to identify any accessions of either barley or wheat able to prevent colonization by *P. graminis*, although certain varieties of oat (*Avena sativa*) and a few *Hordeum* spp. can do so (Barr, 1979; Adams & Jacquier, 1994; Ward et al., 2005; Jin et al., 2022). The present experiments confirmed that *P. graminis* readily colonized all of the barley varieties tested, whether or not they were resistant to JSBWMV, demonstrating that JSBWMV resistance acts against the virus rather than against the vector. A similar situation pertains with resistance to *wheat yellow mosaic virus* (WYMV) in wheat, where two dominant WYMV resistance genes (*Ym1* and *Ym2*) are known (Suzuki et al., 2015; Jiang et al., 2020), the product of which are active in root, but not in leaf, tissue (Liu et al., 2016; Mishina et al., 2023). Two recessively acting WYMV resistance genes, one an allele of *PDIL5-1* and the other of a gene encoding m6A methyltransferase B, have also been identified (Kan et al., 2022; Zhang et al., 2022; Kan et al., 2023); alleles of the former gene in barley (*rym1* and *rym11*) are responsible for conferring resistance against *barley yellow mosaic virus* (Yang et al., 2014), while members of the *rym4/rym5/rym6/rym10* gene cluster each represent a variant allele of *eIF4E* (Stein et al., 2005). As the activity of the products of these resistance genes has to date only been investigated in leaf tissue, it is not known as yet whether any of them act in root tissue.

### The mechanistic basis of genetic resistance against JSBWMV

The present study was undertaken to determine whether *Jmv1* and/or *Jmv2* act in the roots to protect the host from infection. The susceptible variety cv. Tochinoibuki lacks not only both these two major effect genes but also any discernible genes of minor effect (Okada et al., 2022). It allowed the viral titre in its roots to build up to a detectable level very early in the plant’s life cycle; the minor plant-to-plant variation observed with respect to the temporal profile of the virus’ build-up most likely reflects differences between the timing of when the host plants were first colonized by *P. graminis* or perhaps in differences between the timing of the movement of the virus from the vector into the host. The viral titre in the leaf rose above the level of detection during January and peaked in February. Unlike cv. Tochinoibuki, cv. Sukai Golden harbors both *Jmv1* and *Jmv2*, which act to inhibit the build-up of JSBWMV *in planta* (Okada et al., 2022). The delayed appearance of virus in the roots of cv. Sukai Golden implies that the product of at least one of the two major resistance genes is functional in the roots in the early phase of the plant’s life cycle. The difference in viral titre between plants of cv. Tochinoibuki and cv. Sukai Golden persisted until flowering and maturity, which means that either or both of the *Jmv1* and *Jmv2* products continued to inhibit viral multiplication throughout the plants’ life cycle. Nevertheless, the viral titre in cv. Sukai Golden roots was higher than in those of plants never exposed to JSBWMV, showing that the genetic protection harbored by cv. Sukai Golden was not absolute. The implication is that, in cv. Sukai Golden, the virus is inhibited from moving out of the vector into the host root tissue, although it is also possible that the resistance acts to limit the multiplication of virus particles which have succeeded in moving out of the vector. It should be noted, in contrast, that the genetic resistance to WYMV harbored by the wheat variety cv. Madsen is strong enough to exclude any significant colonization of the host by the virus (Mishina et al., 2023). At present, it has not been established whether the JSBWMV present in the extracts of the roots of cv. Sukai Golden and cv. Haruna Nijo remains within the vector or whether at least some of the particles managed to colonize the host plants’ roots. The implication is, however, that the mode of action of JSBWMV resistance is distinct from that of WYMV.

The resistance of cv. Haruna Nijo is due to the presence of a gene mapping to the distal end of the short arm of chromosome 2H, which is presumed to be either identical with or represents an allele of *Jmv1* (Okada et al., 2020; Okada et al., 2022). This cultivar is overall less resistant than the essentially immune cv. Sukai Golden (Okada et al., 2022), probably because it lacks *Jmv2*, the product of which acts to suppress the ability of the virus to multiply in its host’s roots.

### Potential allelic variation at *Jmv1*

The wild barley accession H602 demonstrates a moderate level of JSBWMV resistance (Okada et al., 2020). The genetic basis of this effect is most likely the presence of an alternative allele at *Jmv1* because a genetic study has shown that the resistance of accession H602 could not be satisfactorily explained by the presence of either a minor QTL mapping to the long arm of chromosome 2H or of a second one mapping to the long arm of chromosome 3H (Okada et al., 2020). The infection rate of accession H602 plants varied much more widely across replicates than that of cv. Tochinoibuki plants. The proposal here is that the resistance gene harbored by accession H602 is expressed in a stochastic fashion, resulting in some plants exhibiting a higher level of resistance than others. The mean and plant-to-plant variation for JSBWMV titre were comparable between cv. Haruna Nijo and accession H602 plants over the period December through February, a time when the virus was well able to multiply in a susceptible host such as cv. Tochinoibuki. However, after February, most of the cv. Haruna Nijo plants remained relatively free of the virus, whereas about half of those of accession H602 carried a high viral load. This result implies that the *Jmv1* allele harbored by cv. Haruna Nijo acts constitutively, while the one harbored by accession H602 is more labile.

### Translocation of the virus from the root to the leaf

For each of the three non-immune hosts, the observable presence of virus in the root (viral titre >2) was accompanied by a comparable titre in the leaf. Thus, the translocation of the virus from the root to the shoot was concluded to be largely dependent on a sufficient titre developing in the root. It is therefore unlikely that the gene product of *Jmv1* has any influence over the translocation process, but no such conclusion can be drawn for that of *Jmv2* because the carrier of this gene was immune. As a result, the quantity of virus present in the leaf materials sampled later in the plant life cycle (during March and April) must reflect the presence/absence of resistance mechanisms acting only in the roots.

### An effective qRT-PCR assay for the presence of JSBWMV

The quantitative assay JSBWMV described here suggests that the translocation of virus from the root to the shoot appears to be largely dependent on a sufficient titre developing in the root. The assay proved to be both sensitive and stable. It was able to highlight genotypic differences in the accumulation of JSBWMV in various parts of the host plant, which allowed for direct comparisons to be drawn between the viral titre present across time and space. Its application was able to characterize the process of JSBWMV infection, multiplication, and translocation from the root to the shoot and to identify at what point in time and space in the host’s life cycle the two known major resistance genes are functional.

## Data availability statement

The datasets presented in this study can be found in online repositories. The names of the repository/repositories and accession number(s) can be found in the article/**Supplementary Material**.

## Author contributions

KO, TKa, TKo, and KN conceived the research, KO and KM carried out the microscopic analysis, KO, KM, YO, and KN carried out the qRT-PCR experiments, KO, WX, KM, and TKo the analyzed data, and KO, WX, and TKo wrote the paper. All authors contributed to the article and approved the submitted version.

## Supplementary material

Supplementary Figure 1 Standard curves used for the absolute quantification of **(A)** *P. graminis* rDNA ITS, **(B)** barley *Actin*, **(C)** JSBWMV, and **(D)** barley *Actin* transcript. The PCR efficiencies were estimated as 97.5%, 92.1%, 97.2%, and 97.6%, respectively. The mean Cq (Ct) value obtained from two technical replicates was plotted against log_10_ (sequence copy number per reaction).

## References

[B1] Adams M. J. Jacquier C. (1994). Infection of cereals and grasses by isolates of *Polymyxa graminis* (Plasmodiophorales). Ann. Appl. Biol. 125, 53–60. doi: 10.1111/j.1744-7348.1994.tb04946.x

[B2] Barr D. J. S. (1979). Morphology and host range of *Polymyxa graminis*, *Polymyxa betae*, and *Ligniera pilorum* from Ontario and some other areas. Can. J. Plant Pathol. 1, 85–94. doi: 10.1080/07060667909501468

[B3] Bayles R. O'Sullivan D. Lea V. Freeman S. Henry C. (2007). “Controlling soil-borne cereal mosaic virus in the UK by developing resistant wheat cultivars,” in Home-Grown Cereals Authority (HGCA) project 2616 HGCA crop research news project report. Home-Grown Cereals Authority (HGCA) project, 418. Available at: https://projectblue.blob.core.windows.net/media/Default/Research%20Papers/Cereals%20and%20Oilseed/pr418.pdf.

[B4] Brakke M. K. Langenberg W. G. (1988). “Experiences with soil-borne wheat mosaic virus in north America,” in Developments in applied biology II(Wellesbourne), 183–202.

[B5] Braselton J. P. (1995). Current status of the *plasmodiophorids* . Crit. Rev. Microbiol. 21, 263–275. doi: 10.3109/10408419509113543 8688155

[B6] Cadle-Davidson L. Sorrells M. E. Gray S. M. Bergstrom G. C. (2006). Identification of small grains genotypes resistant to *Soilborne wheat mosaic virus* . Plant Dis. 90, 1039–1044. doi: 10.1094/PD-90-1039 30781296

[B7] Campbell R. N. (1996). Fungal transmission of plant viruses. Annu. Rev. Phytopathol. 34, 87–108. doi: 10.1146/annurev.phyto.34.1.87 15012536

[B8] Clover G. R. G. Ratti C. Henry C. M. (2001). Molecular characterization and detection of European isolates of *Soil-borne wheat mosaic virus* . Plant Pathol. 50, 761–767. doi: 10.1046/j.1365-3059.2001.00634.x

[B9] Hao Y. Wang Y. Chen Z. Bland D. Li S. Brown-Guedira G.. (2012). A conserved locus conditioning *Soil-borne wheat mosaic virus* resistance on the long arm of chromosome 5D in common wheat. Mol. Breed. 30, 1453–1464. doi: 10.1007/s11032-012-9731-x

[B10] Hou J. Liu Q. Q. Xu M. L. (2012). Molecular mechanism of plant defense against virus attack. Acta Agronom Sin. 38, 761–772. doi: 10.3724/SP.J.1006.2012.00761

[B11] Jiang C. Kan J. Ordon F. Perovic D. Yang P. (2020). Bymovirus-induced yellow mosaic diseases in barley and wheat: viruses, genetic resistances and functional aspects. Theor. Appl. Genet. 133, 1623–1640. doi: 10.1007/s00122-020-03555-7 32008056

[B12] Jin Y. Chen S. Xu X. Jiang C. He Z. Shen H.. (2022). Host specificity of soil-borne pathogens in hordeum species and their relatives. Plant Dis. doi: 10.1094/PDIS-04-22-0760-RE 36089682

[B13] Kan J. Cai Y. Cheng C. Chen S. Jiang C. He Z.. (2023). CRISPR/Cas9-guided knockout of *eIF4E* improves *Wheat yellow mosaic virus* resistance without yield penalty. Plant Biotechnol. J. doi: 10.1111/pbi.14002 PMC10106853 36628413

[B14] Kan J. Cai Y. Cheng C. Jiang C. Jin Y. Yang P. (2022). Simultaneous editing of host factor gene *TaPDIL5-1* homoeoalleles confers *Wheat yellow mosaic virus* resistance in hexaploid wheat. New Phytol. 234, 340–344. doi: 10.1111/nph.18002 35092005

[B15] Kanyuka K. Ward E. Adams M. J. (2003). *Polymyxa graminis* and the cereal viruses it transmits: a research challenge. Mol. Plant Pathol. 4, 393–406. doi: 10.1046/j.1364-3703.2003.00177.x 20569399

[B16] Kuhne T. (2009). Soil-borne viruses affecting cereals: known for long but still a threat. Virus Res. 141, 174–183. doi: 10.1016/j.virusres.2008.05.019 19159654

[B17] Liu C. Suzuki T. Mishina K. Habekuss A. Komatsuda T. (2016). *Wheat yellow mosaic virus* resistance in wheat cultivar Madsen acts in roots but not in leaves. J. Gen. Plant Pathol. 82, 261–267. doi: 10.1007/s10327-016-0674-7

[B18] Lyons R. Yilmaz N. D. Powers S. Hammond-Kosack K. E. Kanyuka K. (2009). Characterization of two unusual features of resistance to *Soilborne cereal mosaic virus* in hexaploid wheat: leakiness and gradual elimination of viral coat protein from infected root tissues. Mol. Plant Microbe Interact. 22, 560–574. doi: 10.1094/MPMI-22-5-0560 19348574

[B19] Maccaferri M. Ratti C. Rubies-Autonell C. Vallega V. Demontis A. Stefanelli S.. (2011). Resistance to *Soil-borne cereal mosaic virus* in durum wheat is controlled by a major QTL on chromosome arm 2BS and minor loci. Theor. Appl. Genet. 123, 527–544. doi: 10.1007/s00122-011-1605-9 21594676

[B20] Mishina K. Suzuki T. Oono Y. Yamashita Y. Zhu H. Ogawa T.. (2023). Wheat *Ym2* is oriented from *Aegilops sharonensis* and conferred resistance to soil borne *Wheat yellow mosaic virus* infection to the roots. P Natl. Acad. Sci. U.S.A. doi: 10.1073/pnas.2214968120 PMC10089197 36897977

[B21] Narasimhamoorthy B. Gill B. S. Fritz A. K. Nelson J. C. Brown-Guedira G. L. (2006). Advanced backcross QTL analysis of a hard winter wheat x synthetic wheat population. Theor. Appl. Genet. 112, 787–796. doi: 10.1007/s00122-005-0159-0 16463062

[B22] Ohki T. Sasaya T. Maoka T. (2019). Cylindrical inclusion protein of *Wheat yellow mosaic virus* is involved in differential infection of wheat cultivars. Phytopathology 109, 1475–1480. doi: 10.1094/PHYTO-11-18-0438-R 30951441

[B23] Ohki T. Sasaya T. Sayama M. Maoka T. (2017). Diversity of rDNA-ITS sequences of *Polymyxa graminis* from wheat and barley in Japan. J. Gen. Plant Pathol. 83, 226–230. doi: 10.1007/s10327-017-0713-z

[B24] Okada K. Kato T. Oikawa T. Komatsuda T. Namai K. (2020). A genetic analysis of the resistance in barley to *Soil-borne wheat mosaic virus* . Breed Sci. 70, 617–622. doi: 10.1270/jsbbs.20071 33603558 PMC7878938

[B25] Okada K. Tanaka T. Fukuoka S. Oono Y. Mishina K. Oikawa T.. (2022). Two dominant genes in barley (*Hordeum vulgare* l.) complementarily encode perfect resistance to *Japanese soil-borne wheat mosaic virus* . Breed Sci. 72, 372–382. doi: 10.1270/jsbbs.22046 36776442 PMC9895801

[B26] Russo M. A. Ficco D. B. M. Marone D. Vita P. D. Vallega V. Rubies-Autonell C.. (2012). A major QTL for resistance to *Soil-borne cereal mosaic virus* derived from an old Italian durum wheat cultivar. J. Plant Interact. 7, 290–300. doi: 10.1080/17429145.2011.640437

[B27] Shirako S. Brakke M. K. (1984). Two purified RNAs of *Soil-borne wheat mosaic virus* are needed for infection. J. Gen. Virol. 65, 119–127. doi: 10.1099/0022-1317-65-1-119

[B28] Shirako Y. Suzuki N. French R. C. (2000). Similarity and divergence among viruses in the genus *Furovirus* . Virology 270, 201–207. doi: 10.1006/viro.2000.0251 10772992

[B29] Singer A. C. Crowley D. E. Thompson I. P. (2003). Secondary plant metabolites in phytoremediation and biotransformation. Trends Biotechnol. 21, 123–130. doi: 10.1016/S0167-7799(02)00041-0 12628369

[B30] Soosaar J. L. Burch-Smith T. M. Dinesh-Kumar S. P. (2005). Mechanisms of plant resistance to viruses. Nat. Rev. Microbiol. 3, 789–798. doi: 10.1038/nrmicro1239 16132037

[B31] Stein N. Perovic D. Kumlehn J. Pellio B. Stracke S. Streng S.. (2005). The eukaryotic translation initiation factor 4E confers multiallelic recessive *Bymovirus* resistance in *Hordeum vulgare* (L.). Plant J. 42, 912–922. doi: 10.1111/j.1365-313X.2005.02424.x 15941403

[B32] Suzuki T. Murai M. N. Hayashi T. Nasuda S. Yoshimura Y. Komatsuda T. (2015). Resistance to *Wheat yellow mosaic virus* in Madsen wheat is controlled by two major complementary QTLs. Theor. Appl. Genet. 128, 1569–1578. doi: 10.1007/s00122-015-2532-y 25957645

[B33] Takayama T. Sotome M. Oozeki N. Haruyama M. Yamaguchi T. Okiyama T.. (2011). Breeding of a new two-rowed pearling barley cultivar "Tochinoibuki". Bull. Tochigi Agr Exp. Stn 66, 53–66. Available at: https://www.pref.tochigi.lg.jp/g59/documents/kenpou66-07tochinoibuki.pdf.

[B34] Taniguchi Y. Oda S. Tsunemi J. Ohtsuka M. Sekiwa T. Kumekawa T.. (2001). New two-rowed malting barley cultivar "Sukai golden". Bull. Tochigi Agr Exp. Stn 50, 1–18. Available at: https://www.agrinet.pref.tochigi.lg.jp/nousi/kenpou/kp_050/kp_050_01.pdf.

[B35] Ward E. Kanyuka K. Motteram J. Kornyukhin D. Adams M. J. (2005). The use of conventional and quantitative real-time PCR assays for *Polymyxa graminis* to examine host plant resistance, inoculum levels and intraspecific variation. New Phytol. 165, 875–885. doi: 10.1111/j.1469-8137.2004.01291.x 15720699

[B36] Xu Y. Hu L. Li L. Zhang Y. Sun B. Meng X.. (2018). Ribotypes of *Polymyxa graminis* in wheat samples infected with soilborne wheat viruses in China. Plant Dis. 102, 948–954. doi: 10.1094/PDIS-09-17-1394-RE 30673393

[B37] Yang P. Habekuss A. Ordon F. Stein N. (2014). Analysis of bymovirus resistance genes on proximal barley chromosome 4HL provides the basis for precision breeding for BaMMV/BaYMV resistance. Theor. Appl. Genet. 127, 1625–1634. doi: 10.1007/s00122-014-2324-9 24849455

[B38] Ye R. Zheng T. Chen J. Diao A. Adams M. J. Yu S.. (1999). Characterization and partial sequence of a new *Furovirus* of wheat in China. Plant Pathol. 48, 379–387. doi: 10.1046/j.1365-3059.1999.00358.x

[B39] Zhang T. Shi C. Hu H. Zhang Z. Wang Z. Chen Z.. (2022). N6-methyladenosine RNA modification promotes viral genomic RNA stability and infection. Nat. Commun. 13, 6576. doi: 10.1038/s41467-022-34362-x 36323720 PMC9629889

