# Supplementary figures and images for: Genetic resistance in barley against Japanese soil-borne wheat mosaic virus functions in the roots
Source: Front Plant Sci. 2023 Mar 10;14:1149752. doi: 10.3389/fpls.2023.1149752 (PMC10036763; doi:10.3389/fpls.2023.1149752)

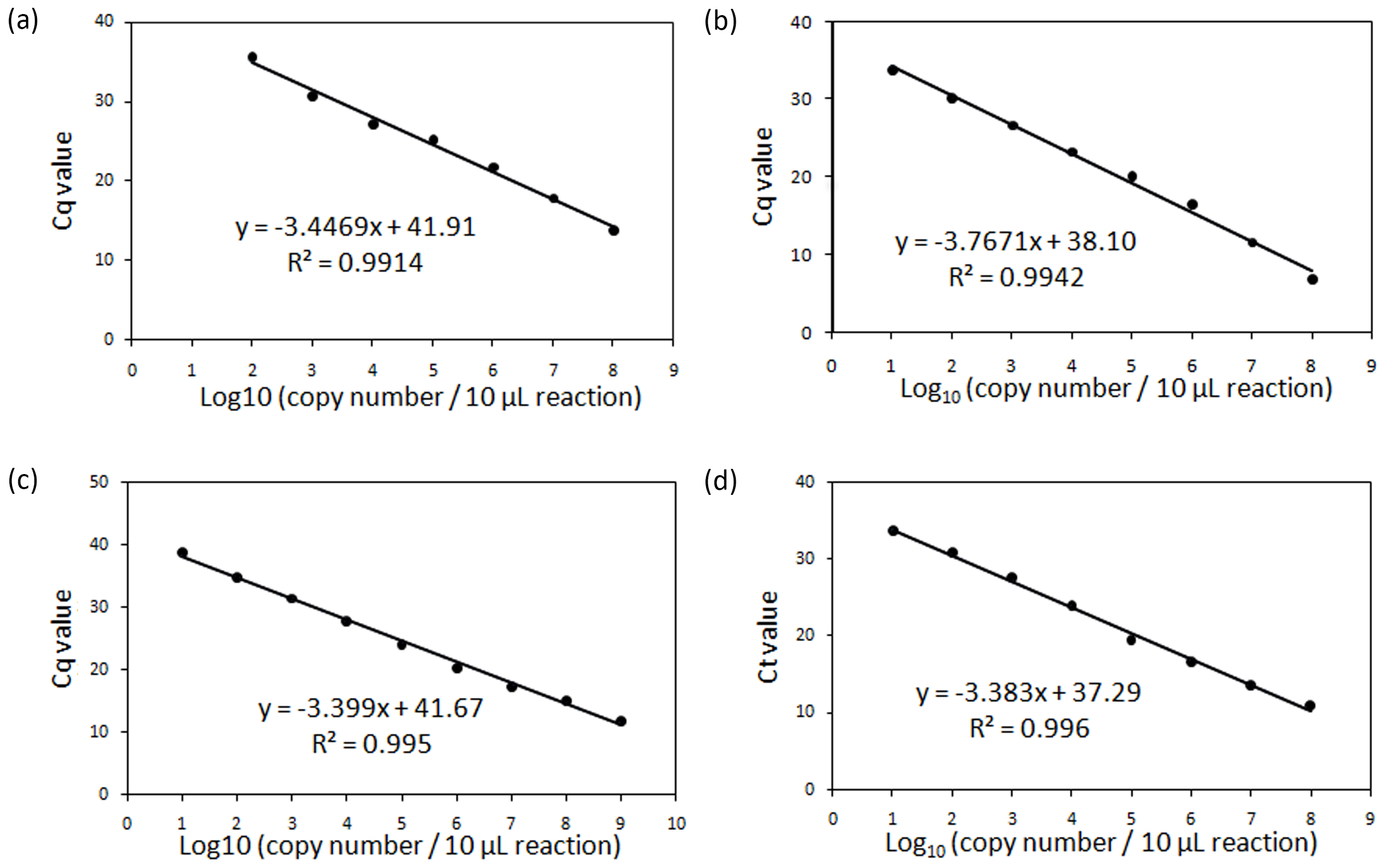

Supplement: Supplementary Figure 1 — Standard curves used for the absolute quantification of (A) P. graminis rDNA ITS, (B) barley Actin, (C) JSBWMV, and (D) barley Actin transcript. The PCR efficiencies were estimated as 97.5%, 92.1%, 97.2%, and 97.6%, respectively. The mean Cq (Ct) value obtained from two technical replicates was plotted against log10 (sequence copy number per reaction). [file Image_1.jpeg]
